# Galectin-3C Inhibits Tumor Growth and Increases the Anticancer Activity of Bortezomib in a Murine Model of Human Multiple Myeloma

**DOI:** 10.1371/journal.pone.0021811

**Published:** 2011-07-13

**Authors:** Leonardo Mirandola, Yuefei Yu, Kitty Chui, Marjorie R. Jenkins, Everardo Cobos, Constance M. John, Maurizio Chiriva-Internati

**Affiliations:** 1 Division of Hematology & Oncology, Texas Tech University Health Sciences Center and Southwest Cancer Treatment and Research Center, Lubbock, Texas, United States of America; 2 Laura W. Bush Institute for Women's Health and Center for Women's Health and Gender-Based Medicine, Texas Tech University Health Sciences Center, Amarillo, Texas, United States of America; 3 MandalMed, San Francisco, California, United States of America; Vanderbilt University Medical Center, United States of America

## Abstract

Galectin-3 is a human lectin involved in many cellular processes including differentiation, apoptosis, angiogenesis, neoplastic transformation, and metastasis. We evaluated galectin-3C, an *N*-terminally truncated form of galectin-3 that is thought to act as a dominant negative inhibitor, as a potential treatment for multiple myeloma (MM). Galectin-3 was expressed at varying levels by all 9 human MM cell lines tested. *In vitro* galectin-3C exhibited modest anti-proliferative effects on MM cells and inhibited chemotaxis and invasion of U266 MM cells induced by stromal cell-derived factor (SDF)-1α. Galectin-3C facilitated the anticancer activity of bortezomib, a proteasome inhibitor approved by the FDA for MM treatment. Galectin-3C and bortezomib also synergistically inhibited MM-induced angiogenesis activity *in vitro*. Delivery of galectin-3C intravenously via an osmotic pump in a subcutaneous U266 cell NOD/SCID mouse model of MM significantly inhibited tumor growth. The average tumor volume of bortezomib-treated animals was 19.6% and of galectin-3C treated animals was 13.5% of the average volume of the untreated controls at day 35. The maximal effect was obtained with the combination of galectin-3C with bortezomib that afforded a reduction of 94% in the mean tumor volume compared to the untreated controls at day 35. In conclusion, this is the first study to show that inhibition of galectin-3 is efficacious in a murine model of human MM. Our results demonstrated that galectin-3C alone was efficacious in a xenograft mouse model of human MM, and that it enhanced the anti-tumor activity of bortezomib *in vitro* and *in vivo*. These data provide the rationale for continued testing of galectin-3C towards initiation of clinical trials for treatment of MM.

## Introduction

Multiple myeloma (MM) is a malignancy characterized by clonal proliferation and accumulation of terminally differentiated plasma cells that produce immunoglobulin (Ig). The malignant plasma cells are found in the bone marrow and extramedullary locations. The American Cancer Society estimated that there were more than 20,000 new cases of MM in the US in 2010. Currently chemotherapy induces complete tumor regression in 50% of patients but the median survival of MM is only about 5 years [1]. The introduction of novel drugs such as thalidomide, lenalidomide, and bortezomib (Bor) that are thought to target specific intracellular pathways and affect cellular interactions with the tumor microenvironment, have aided in the treatment of MM especially in management of elderly patients [2]. Nonetheless, arguably the course of the disease has not fundamentally changed since the 1960s.

During the last ten years, intriguing insights into the molecular mechanisms underlying the progression of MM have been obtained and successfully translated into more effective therapeutics, such as the proteasome inhibitors [3]. Bor, a boronic acid dipeptide, was the first-in-class proteasome inhibitor approved by the US Food and Drug Administration (FDA) for the treatment of relapsed and refractory MM [4]–[6]. Deregulation of the ubiquitin-proteasome signaling pathway is linked to the etiology of various human diseases and, therefore, proteasome inhibitors offer great promise as therapeutic agents [7], [8].

A human carbohydrate-binding protein that may play a role in MM is galectin-3, a member of the galectin family that is defined based on sequence homology within the carbohydrate recognition domain (CRD) and a characteristic affinity for β-galactosides [9]. Galectin-3 can be variously located either intra- or extracellularly, and is unique in the galectin family because in addition to the carboxy-terminal CRD [10]–[12], it has an amino-terminal domain that is critical for multivalent behavior. Alone, the carboxy-terminal CRD has been reported to lack hemagglutination activity and cooperative binding that are characteristics of the intact lectin [13]. The *N*-terminal domain enables the CRD to cross-link carbohydrate-containing ligands on cell surfaces and in the extracellular matrix and, thus, to modulate cell adhesion and signaling [14]. There is striking evidence that galectin-3 plays a role in neoplastic transformation, tumor growth, cancer cell adhesion, metastasis, invasion, and apoptosis [15], [16].

The truncated form of galectin-3 (Gal-3C) used in this study consists of 143 carboxy-terminal amino acid residues of human galectin-3. Gal-3C retains carbohydrate binding ability but lacks the *N*-terminal domain and, therefore, is expected to act as a dominant negative inhibitor of galectin-3 carbohydrate binding and subsequent homophilic cross-linking mediated by the *N*-terminal domain that promotes cell adhesion and consequent survival signals [17].

The proteasome inhibitor Bor frequently becomes ineffective in treatment of MM due to the development of drug resistance and the drug also displays significant off-target toxicities [18]. In an attempt to overcome these difficulties and to assess the potential of Gal-3C as a treatment for MM, the present study evaluated the efficacy of Gal-3C alone, and in combination with Bor in NOD/SCID immunodeficient mice implanted with human MM cells.

We found that Gal-3C inhibited the growth of MM U266 tumors in a subcutaneous murine model with activity that was greater than Bor, and that combination of Gal-3C with Bor further inhibited tumor growth. In addition, we show that *in vitro* Gal-3C and Bor can synergistically inhibit MM-induced angiogenesis.

## Results

### Galectin-3 is expressed by MM cell lines

**Figure 1:**
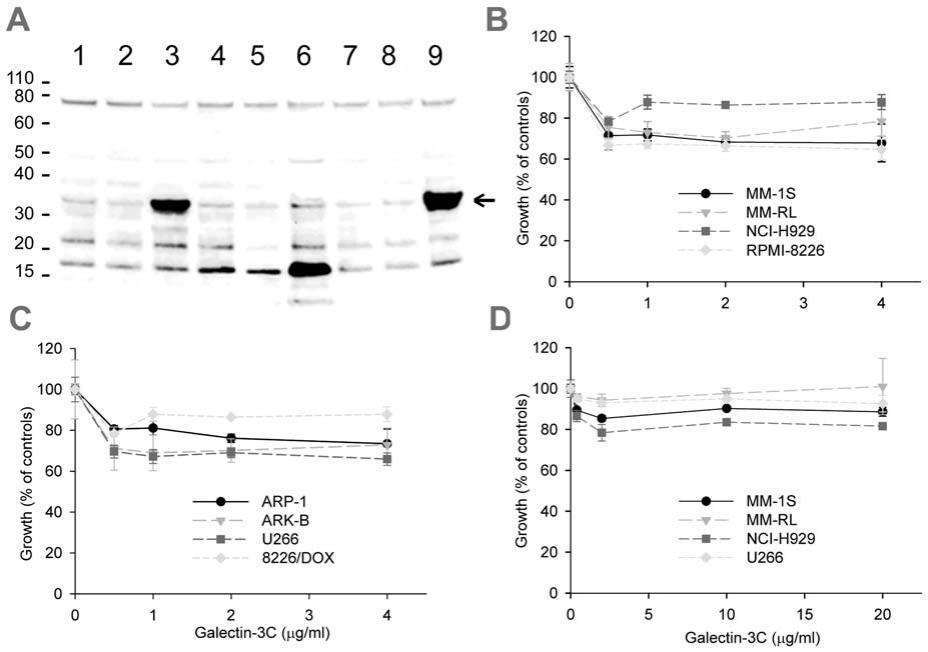
Galectin-3 expression levels in MM cell lines and effects of galectin-3 inhibition *in vitro.* (**a**) The lysates in each lane are as follows: 1 MM-1S; 2 MM-1RL; 3 NCI-H929; 4 RPMI-8226; 5 Dox-40; 6 LR-5; 7 ARP-1; 8 ARB-B; 9 U266. Indicated MM cell lines were seeded at 300,000 cells/mL and maintained at 37°C and 5% CO_2_. Total protein fractions from exponentially growing cells were prepared and analyzed by immunoblot to detect galectin-3. Galectin-3 monomers are approximately 30 kDa (arrow). Detection of bands at higher and lower masses is consistent with reports of the multimerization of the protein and its digestion by matrix metalloproteinases. (**b**) Graph of the results from proliferation assays shows the relative responses of MM-1S, MM-RL, NCI-H929, and RMPI-8266 and, (**c**) ARP-1, ARK-B, U266, and 8226/Dox cells to 0.5, 1, 2, or 4 µg/ml Gal-3C compared to control wells over 48 h. There was a significant decrease in proliferation in response to Gal-3C treatment (defined as *P*<0.05; *t*-test) in all eight cell lines at one or more concentrations 48 h. Triplicate data points were analyzed and the data shown are representative of 3 or more experiments. Error bars are±SD. (**d**) Graph of the results from proliferation assays with MM-1S, MM-RL, NCI-H929, and U266 showing the relative response to 0.4, 2, 10,or 20 µg/ml Gal-3C compared to control wells over 24 h. There was a significant decrease (*P*<0.01) in proliferation of MM-1S, NCI-H929, and U266 cells in response to Gal-3C at one or more concentration after 24 h. Error bars are±S.D.

Proteins derived from a panel of 9 human MM cell lines, namely MM-1S, MM-1RL, NCI-H929, RPMI-8226, 8226/Dox-40, 8226/LR-5, ARP-1, ARK-B, and U266, were analyzed by Western blot to detect galectin-3 expression. Figure 1a indicates that all of the cell lines expressed galectin-3 (∼30 kDa). Expression levels of monomeric galectin-3 in the cells differed, with U266 and NCI-H929 having the highest levels.

The M3/38 anti-galectin-3 antibody also stained three other bands that were present in most or all of the samples. Previous studies indicate that this antibody binds to the *N*-terminal domain of the full-length protein or to an *N*-terminal fragment of it [19]. Bands for two smaller proteins can be observed with one somewhat greater than 20 kDa and the second at about 17 kDa. Galectin-3 is subject to digestion by mammalian metalloproteinases (MMPs) including MMP-2, -9, and -13 that primarily cleave the Ala_62_ –Tyr_63_ bond yielding a fragment of ∼22 kDa containing the CRD and an *N*-terminal fragment [20], [21]. The Gly_32_-Ala_33_ bond is an alternate site for cleavage by MMPs that yields a 27 kDa fragment containing the CRD [22].

Another band that appears at approximately 75 kDa in size is of similar intensity in each sample. Galectin-3 can form non-covalent homodimers and higher order multimers by binding mediated by its *N*-terminal domain [23], and can form covalent homodimers through intermolecular disulfide bonds of cysteine [24], and through reactivity with tissue transglutaminase that catalyzes the cross-linking of glutamine with other amino acids [25], [26]. Prior to electrophoresis, the samples were heated at 70 °C rather than boiled, thus, it is also possible that the 75 kDa bands may be composed of non-covalent homodimers of galectin-3. Transglutamination should not be affected by reduction, but it is unlikely that disulfide bonds would have survived the treatment of the samples with the reducing agent, 2-mercaptoethanol.

### Gal-3C inhibited the proliferation of MM cells

MM cell lines were plated in 96-well plates, Gal-3C was added, and the relative proliferation determined after incubation of the plates for either 24 or 48 h. Results from representative analyses are shown in Figure 1b-d. Gal-3C inhibited the proliferation of all eight cell lines at the 0.5, 1, 2, or 4 µg/ml concentration (b-c) after 48 h (*P*<0.05). Gal-3C treatment also significantly (*P*<0.01) inhibited the proliferation of MM-1S, NCI-H929, and U266 but not the MM-RL cell line at 0.4, 2.0, 10, or 20 µg/ml concentrations at 24 h. The overall magnitude of the effects observed with any of the cell lines was slight.

### Chemotaxis of U266 MM cells was reduced by Gal-3C

**Figure 2:**
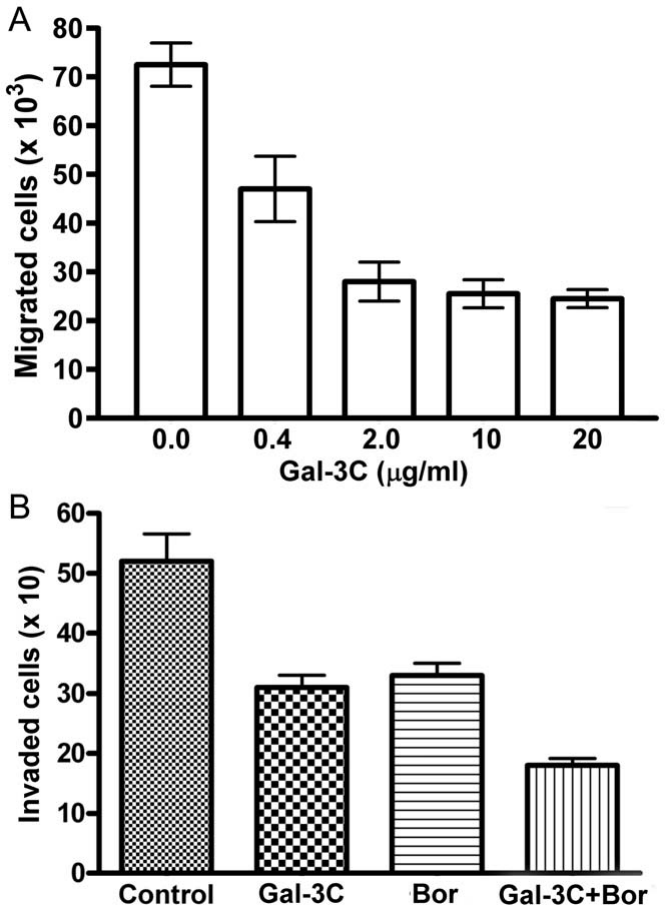
Gal-3C inhibits the chemotaxis and invasion of U266 MM cells. (**a**) Gal-3C (0.4, 2, 10, or 20 µg/ml) reduces the chemotaxis of U266 cells in bicameral chambers in response to SDF-1α (100 ng/ml). Error bars are±S.D. and sextuplicate data points are represented (ANOVA with Tukey test *P*<0.001 for all compared to controls). (**b**) The effect of Gal-3C and Bor on invasion activity of U266 cells. After starvation in serum-free medium for 3 h, U266 cells were plated on Matrigel-coated inserts in the upper chambers. The number of cells which migrated through the Matrigel and filter from the upper chamber and were adhering to the bottom of the insert were quantified by counting using a microscope. Gal-3C (10 µg/ml) and Bor (5 nM), and the combination of both significantly inhibited (*P*<0.05) U266 cell invasion. Experiments were done in triplicate and repeated three times. Error bars represent ±SD.

The addition of Gal-3C to the media with U266 cells in the top chambers of bicameral transwell chambers (Figure 2a) significantly (*P*<0.001 for all Gal-3C treatments compared to controls) reduced the migration of the cells into the bottom chamber containing SDF-1α, a potent lymphocyte chemotactic factor [27]. The percentage of inhibition increased by more than 70% using 2.0 µg/ml Gal-3C compared to a 0.4 µg/ml concentration. However, increasing the concentration to 10 and 20 µg/ml produced only a slightly higher percentage of inhibition compared to 2.0 µg/ml.

### Gal-3C inhibited the invasion of U266 cells and facilitated Bor-mediated inhibition of invasion

To assess the ability of Gal-3C and Bor to inhibit the invasion of MM cells, an assay was performed in 24-well Transwell® chamber plates with 6.5-mm inserts containing polycarbonate membranes with 5 µm pores coated with 50 µl Matrigel™ (Becton Dickinson, Bedford, MA, USA) [28]. After serum starvation for 3 h, U266 cells (5×10^4^) were added to the upper chambers with and without Gal-3C (10 µg/ml), or Bor (5 nM), or the combination treatment. To each lower chamber, 600 µl of media was added with 10% FBS and 100 ng/ml SDF-1α. The U266 cells on the bottom of each insert were Coomassie-stained and counted using a microscope after incubating the plates at 37°C for 24 h. The data as shown in Figure 2b revealed that both Gal-3C and Bor treatments significantly decreased the invasion of the U266 cells by more than 30% (*P*<0.05). In addition, there were significantly fewer invaded cells in the wells treated with the combination of Gal-3C plus Bor compared to wells treated with Gal-3C or Bor alone (*P*<0.05). These results show that both Gal-3C and Bor inhibited invasion by the U266 cells *in vitro*, and that the combination treatment was more inhibitory than either alone.

### Gal-3C and Bor synergistically inhibited endothelial cell migration

**Figure 3:**
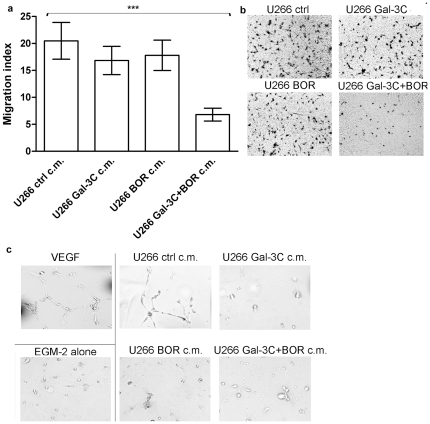
Vascular endothelial cell migration and tubule formation. (**a**) 5×10^4^ serum-starved HUVEC cells were loaded in the upper chamber of a Transwell^®^ plate (8 µm-diameter pores). The bottom chamber was filled with 600 µL serum-free EBM-2 medium supplemented with 20% V/V conditioned (c.m.) derived from U266 cultures undergone different treatments: control (ctrl), Gal-3C, Bor, or Gal-3C and Bor combined (Gal-3C+Bor). EBM-2 medium alone was used as a negative control. After 16-h incubation, chamber filters were removed and migrated cells were fixed and stained with Coomassie brilliant blue as described in Materials and Methods. Mean migration indices (error bars are±SD) were expressed as the number of cells migrated in the presence of the indicated stimulus divided by the number of cells migrated in the presence of EBM-2 alone. One-way ANOVA and Tukey's post-test indicated that Gal-3C+Bor migration index was significantly different from control (*P<0.001), while no significant difference was detected between the control and Gal-3C or Bor alone (*P*>0.05). All assays were run in triplicate. (**b**) representative pictures of the filters. (**c**) 5×10^3^ serum-starved HUVEC were allowed to form tubules on a Matrigel™ surface for 16 h in the presence of VEGF (positive control), EMB-2 medium alone (negative control), or EBM-2 supplemented with 20% V/V U266 c.m. Representative pictures from 3 comparable experiments are shown.

To evaluate the effects of Gal-3C on MM cell-induced migration of endothelial cells, a Transwell®-based chemotaxis assay was performed as follows. U266 cells were cultured in media in the presence of 10 µg/mL Gal-3C, 5 nM Bor, the combined treatment, or vehicle (PBS)-only. After 48 h, conditioned media (c.m.) derived from U266 cells given these different treatments were diluted to 20% with EBM-2 medium and used as a chemoattractant for human umbilical cord vascular endothelial cells (HUVEC) cells. Without considering uptake by the U266 cells, the final concentration of drugs in the HUVEC chemotaxis assay was 2 µg/mL Gal-3C and 1 nM Bor. Results of a 16-h migration assay are displayed in Figure 3a and 3b. Single treatments did not produce a significant variation in the migration index, while the combined treatment resulted in an approximately 70% reduction of the HUVEC migration.

### Gal-3C and Bor inhibited tubule formation in angiogenesis assay

To evaluate the effect of different treatments on the ability of U266 cells to induce angiogenesis, a Matrigel™-based tubule formation assay was performed in the presence of U266 c.m. as described for the chemotaxis assay. Serum-starved (5×10^3^) HUVEC were seeded on the Matrigel surface in 200 µL EBM-2 medium supplemented with 20% V/V c.m. Pictures were taken after incubation of the cells in 5% CO_2_ at 37°C for 16 h. The assay was run in triplicate and representative images are depicted in Figure 3c. As a positive control, EBM-2 medium supplemented with 20 ng/mL VEGF was also tested. Results indicate that treatment with Gal-3C or Bor alone as well as in combination almost completely impaired the potential of U266 cells for induction of angiogenesis, as evidenced by the absence of the organized tubule structures that were detectable only in the control wells with VEGF-treated or untreated U266 cell-c.m.

### Gal-3C did not affect endothelial cell viability

**Figure 4:**
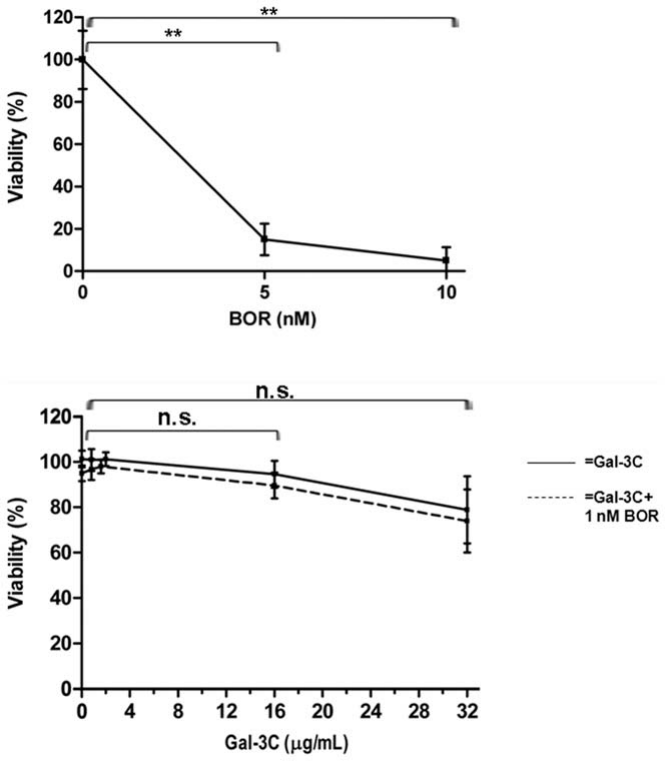
Vascular endothelial cell viability assay. Viability was measured as described for U266 cells in experiments run in triplicate and displayed as percentage of control (untreated) cells. The graphs show mean values and error bars represent±SD. **ANOVA Tukey's post test *P*<0.01. n.s. = not significant.

To determine if the apparent effects of Gal-3C alone or in combination with Bor on vascular endothelial cell migration or secretion were instead due to reduced cell viability, HUVEC cells were treated with Gal-3C, Bor, or both agents for 48 h. Viability was measured as described for the U266 cells. Because at the highest concentrations tested, Gal-3C had a mild effect on HUVEC viability and Bor alone showed dramatic inhibition, lower concentrations of Gal-3C (2 µg/ml) and Bor (1 nM) were used for the chemotaxis and angiogenesis assays. Results obtained from the HUVEC viability experiments that were run in triplicate are depicted in Figure 4, and are presented as a percentage of control (untreated) cells.

### Gal-3C and Bor reduced ανβ3 integrin clustering in HUVEC cells

**Figure 5:**
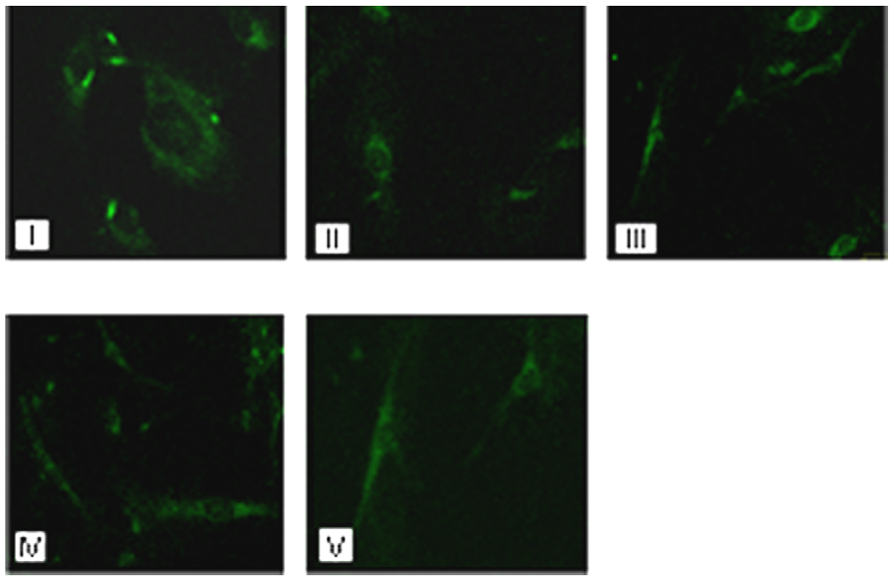
ανβ3 integrin clustering assay. After culturing for 48 h with 20% V/V U266 c.m., HUVEC cells were fixed and stained with anti- αvβ3 antibody. Integrin clusters are evidenced as high density spots in the HUVEC treated with U266 c.m. (I), but not when HUVEC were incubated with c.m. derived from U266 treated with Gal-3C (II), Bor (III), or Gal-3C+Bor (IV). Clusters were also absent in HUVEC treated with EGM-2 medium alone (V). The figure shows representative photomicrographs obtained by confocal microscopy (60X magnification) from three independent experiments.

To evaluate the effects of different treatments on the ability of U266 cells to induce integrin engagement on endothelial cells, HUVEC were incubated with c.m. obtained from U266 cells cultured in 10 µg/mL Gal-3C, 5 nM Bor, or the combination (as described for the chemotaxis assay) for 48 h. Confocal photomicrographs were taken after immunofluorescence staining for ανβ3 integrin. Formation of clusters indicates integrin engagement and it is evidenced as higher fluorescence intensity. Figure 5 shows that Gal-3C and Bor, as well as the combination treatment, were able to block the formation of clusters of ανβ3 integrins that were detected only in wells incubated with c.m. derived from untreated U266 (control) cells. No clusters were evidenced without the addition of c.m. (EGM-2 only).

### Gal-3C and Bor inhibited secretion of VEGF and bFGF by U266 MM cells

**Figure 6:**
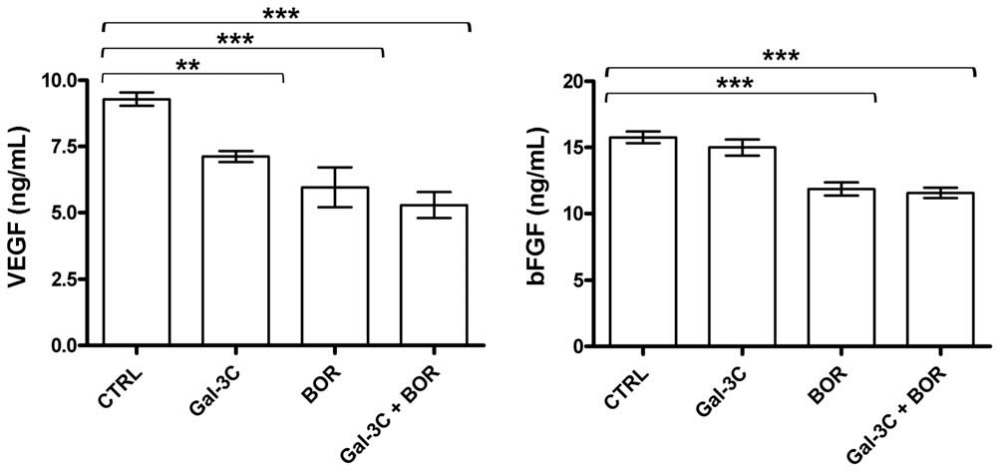
Molecular effects of Gal-3C and Bor on U266 cells. After 48 h in the presence of indicated treatments, the c.m. of U266 cells was diluted 1∶2 in ELISA coating buffer and used in a direct ELISA for the measurement of VEGF and bFGF. Histograms represent mean values of 3 independent experiments. Error bars indicate±SD. Statistical analysis was performed through one-way ANOVA and Tukey's post-test: **P<0.01; ***P<0.001.

A direct enzyme-linked immunoabsorbent assay (ELISA) was performed to measure the levels of VEGF and bFGF in media from U266 cells treated with 10 µg/mL Gal-3C, 5 nM Bor, Gal-3C with Bor, or control media with vehicle (PBS) only for 16 h. The levels of VEGF and bFGF were extrapolated using standard curves obtained by analysis of 10-fold serial dilutions (0-1,000 ng/mL) of the purified recombinant proteins (Abcam, MA, USA). Figure 6 shows the results from 3 independent experiments. Gal-3C alone significantly reduced VEGF but not bFGF levels, while Bor alone or in combination with Gal-3C significantly reduced both VEGF and bFGF levels.

### Generation and testing of AAV/GFP virus stocks

A structural map of the adeno associated virus (AAV)/GFP vector is presented in Fig. S1a. The restriction sites XbaI and NotI were used to insert the GFP gene downstream of the p5 promoter. Panel b illustrates the identification of AAV/GFP by restriction enzyme analysis. GFP was cut from AAV/GFP by XbaI and NotI enzymes. The titer of virus stocks was determined by real-time PCR to be 10^8^ encapsulated genomes (Fig. S1c). After generation of the AAV/GFP virus stock, we evaluated the infection of U266 cells. The AAV/GFP vector-infected cells expressed GFP, as confirmed by cytofluorimetric analysis, RT-PCR and immunofluorescence labeling (Fig. S1d-f).

### Growth of U266 tumors was inhibited by Gal-3C alone and Gal-3C with Bor

**Figure 7:**
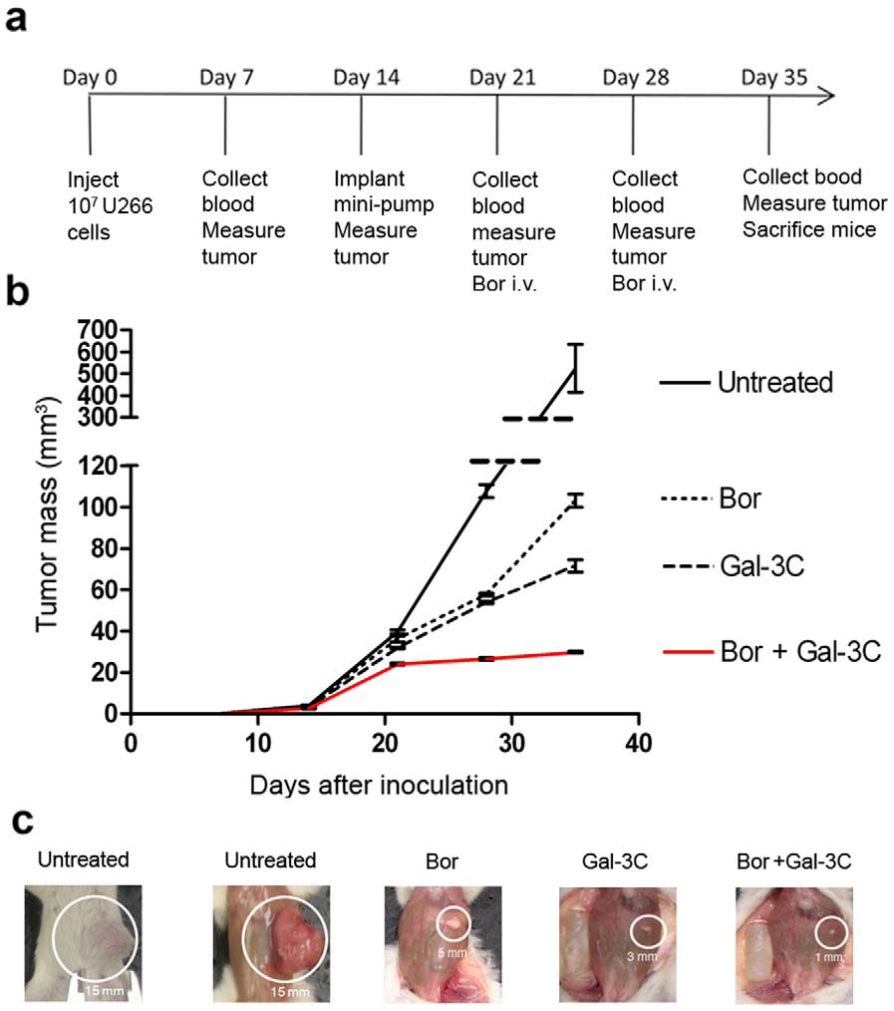
Experimental design and outcome of Gal-3C treatment in NOD/SCID MM xenografts. (**a**) The diagram shows the timing of tumor challenge, drug administration, and sample collections. (**b**) Gal-3C reduced the growth of U266 MM tumors as a single agent, and in combination with Bor significantly reduced the MM tumor growth compared to treatment with either agent alone. The primary tumors were measured weekly with calipers after subcutaneous inoculation of mice with U266 cells. The results represent the combination of 3 separate experiments with n = 5 mice per group in each experiment, and the error bars represent±SEM. The mean tumor volume in the Bor-treated group was significantly less than in the untreated group from d 28 through d 35 (unpaired t test *P*<0.001). The mean tumor volume in the group treated with Gal-3C alone also was significantly less than that in the group treated with Bor alone from d 21 through 35 (unpaired t test *P*<0.001). At d 35, the mean tumor volumes of the Gal-3C-only and the Bor-only group were 13.5% and 19.6% that of the untreated controls, respectively. (**c**) Representative images are presented illustrating the difference between the groups in tumor size after 35 d.

The sequence of procedures used to establish the U266 MM model in the NOD/SCID mice and to treat the mice with Gal-3C, Bor, and the combination of Gal-3C and Bor is shown in Figure 7a. Tumor volumes (Figure 7b) were measured with calipers once weekly. Treatment with vehicle, monotherapy with Gal-3C or Bor, or combination therapy began on d 14.

The average tumor volume in the Bor-treated group was significantly less than in the untreated group from d 28 through d 35 (unpaired t test *P*<0.001), while Gal-3C and Bor+Gal-3C groups displayed tumors significantly smaller than untreated animals (82% and 61%, respectively) as soon as d 21 (unpaired t test *P*<0.001). At d 35, the average tumor volumes of the Gal-3C-only and the Bor-only group were 13.5% and 19.6% that of the untreated controls, respectively.

The average tumor volume in the groups receiving the combination of Gal-3C with Bor was significantly less than that of the groups treated with Bor (or with Gal-3C) beginning on d 21 (unpaired t test *P*<0.001). Gal-3C treatment produced a significant reduction of tumor volume compared with Bor starting on d 21 (unpaired t test *P*<0.001), with a mean difference between the two of 4.431±1.494 mm^3^, through d 35 when the mean difference was 31.54±4.379 mm^3^ (unpaired t test *P*<0.001). The maximum effect was observed on d 35, when the combination therapy afforded a reduction of 94% in the mean tumor volume, compared with the untreated group (unpaired t test *P*<0.001).

**Figure 8:**
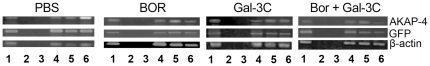
Analysis of AKAP-4 and GFP expression. RT-PCR showing the expression of AKAP-4 and GFP in the tumor masses in the four groups (5 mice/group): (1) U266 (2) no RT (3) no template (4) d 21 (5) d 28 (6) d 35. The U266 cell line was used as a positive control after culture for 48-h.

Specimens of the subcutaneous tumors were analyzed for mRNA encoding AKAP-4 and GFP by RT-PCR weekly. The results (Figure 8) demonstrated the expression of both GFP and AKAP-4 and confirmed that the tumor masses originated from U266 cells. The expression levels of AKAP-4 and GFP in the Gal-3C plus Bor group were lower than any of the other groups at d 35.

**Figure 9:**
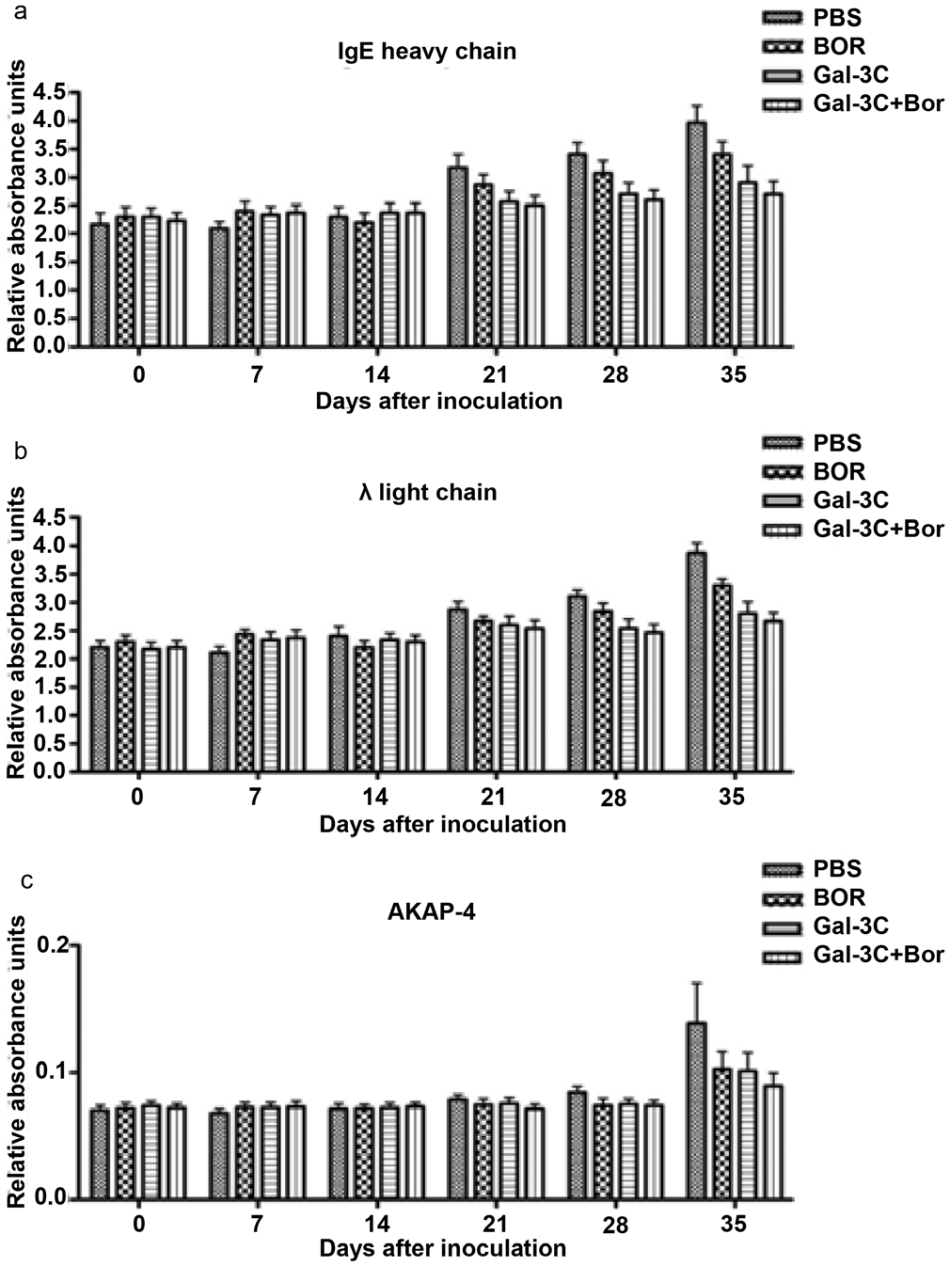
ELISA for human immunoglobulins and AKAP-4 in mouse sera. The mice were bled at the indicated time point after inoculation of U266 cells, and the levels of IgE heavy (A) and light chains (B), and AKAP-4 (C), were determined by ELISA. The levels of heavy and light chain IgE and AKAP-4 were significantly lower in the treated groups than in the PBS group (*P*<0.05) on d 21, 28, and 35, and the levels in the Gal-3C plus Bor animals were the lowest of any group. Each graph is representative of three experiments with samples analyzed in triplicates, and the error bars represent±SD.

AKAP-4 also was detected in the sera of tumor-bearing mice (Figure 9 bottom panel). Tumor growth was associated with increased AKAP-4 levels in the serum beginning in the vehicle-only group on d 21. At 35 d after inoculation, AKAP-4 in the sera from the combination therapy (Gal-3C plus Bor) group was lower than the PBS group.

### Tumor expression of human IgE and Igλ was diminished in treated groups

**Figure 10:**
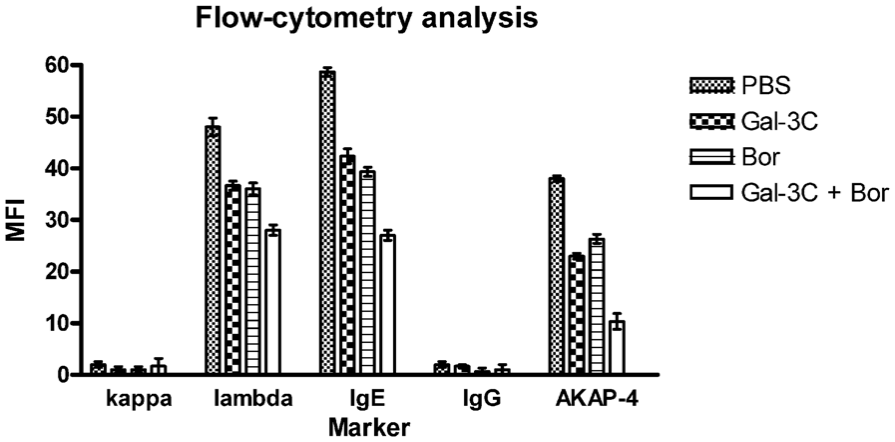
Flow cytometric analyses of the expression of IgE, IgG, Igκ, Igλ, and AKAP-4. Cells from liver, blood, and tumor masses of animals from each group were minced under sterile conditions at RT to obtain single-cell suspensions. Histograms show the mean fluorescence intensity (MFI) and the error bars indicate±SD calculated from 3 independent experiments with samples analyzed in triplicates. The results show that IgE, Igλ, and AKAP-4 were expressed by the tumor cell; Igκ and IgG were expressed at very low levels. Expression levels of IgE, Igλ, and AKAP-4 in the PBS control group were the highest, while the levels in the tumors of the group treated with the combination of Gal-3C with Bor were the lowest.

The presence of human Ig in the sera of the tumor-bearing mice was confirmed by ELISA and flow cytometry (Figures 9 and 10). The tumors expressed human IgE and Igλ, while human IgG and Igκ were very low level in either the tumors or the sera of the mice. Moreover, the expression levels of IgE and Igλ in the treated groups (Figure 9 top and middle panel, respectively) were significantly less (*P*<0.05) than the levels in the controls. The lowest expression levels of IgE and Igλ were observed in the tumors of the group treated with Gal-3C with Bor.

Flow cytometric analyses confirmed the expression of IgE and Igλ by the tumors (Figure 10), and revealed that the highest expression levels of IgE, Igλ and AKAP-4 were in the tumors of the PBS-only control group. The levels of all three were lowest in the group treated with the combination of Gal-3C with Bor, a result that reflected the results from the analysis of the tumor volumes of the groups.

## Discussion

Current treatment for MM is based on high-dose chemotherapy, radiotherapy, and autologous hematopoietic stem cell transplantation. What triggers MM is not well understood and since fatal relapses are routinely observed even after complete remissions, it remains a fatal hematological disease [29].

The proteasome inhibitor Bor was approved for the treatment of relapsed and refractory MM by the FDA in 2003 [30], but dose-limiting toxicities and the development of resistance often prevent its long-term use. Notably, although Bor alone or in combination with other agents, such as thalidomide or lenalidomide, provides up to 85% response rates, nearly 100% of patients eventually relapse [29]. These findings underscore the urgent need to develop new multiple-targeted therapies for more effective treatment of MM.

In the present study, we evaluated the efficacy of monotherapy with Gal-3C, which is an *N*-terminally truncated form of galectin-3, and Gal-3C in a combination therapy with Bor in a NOD/SCID mouse model harboring human MM.

Immunoblot analysis of human MM cell lines revealed that all of the cell lines expressed at least a low level of galectin-3, and that NCI-H929 and U266 cells displayed the highest levels (Figure 1a). The U266 cells were chosen for the animal model because of their relatively high expression level of galectin-3. Varying levels of galectin-3 fragments were observed in the cell lines and were highest in the mephalin-resistant 8226/LR-5 cells.

Galectin-3 is a substrate for MMP digestion. Recent reports have shown that peptides corresponding to galectin-3 fragments formed by MMP cleavage that contained the CRD but which were longer than Gal-3C increased tumor growth and angiogenesis in breast cancer xenograft models [22]. Testing various galectin-3 fragments showed that greater endothelial cell chemotaxis and more capillary-like morphogenesis were induced by peptides corresponding to the *N*-terminal 1–62 amino acids and a molecule containing the CRD corresponding to amino acids 33–250 [31]. Prostate specific antigen (PSA) is a chymotrypsin-like serine protease in human seminal plasma that has been shown to cleave galectin-3 between Y^107^-G^108^ which would yield the Gal-3C of this study, and the corresponding *N*-terminal domain [32], suggesting that Gal-3C is unlikely to be very antigenic in humans.

Our *in vitro* analyses indicated that Gal-3C modestly inhibited the proliferation of all 8 MM cell lines tested. We postulate that this effect on proliferation was due to inhibition of galectin-3 which contains the NWGR domain characteristic of the Bcl-2 family [33] and has been shown to inhibit apoptosis [34] and increase chemoresistance in cancer [35]. However, the sensitivity of the NCI-H929 and U266 cell lines, which had the highest expression levels of monomeric galectin-3, was no greater than that of the other MM cell lines. Also, there was little dose-response effect observed likely due to increased Gal-3C multimerization at higher concentrations. Although self-association through the *N*-terminal domain when galectin-3 is bound to cell surface glycoconjugates has been reported to cause the aggregation of various types of cells [36], [37], lactose-inhibitable homodimerization mediated by the CRD has also been observed [38]. The possibility that the primary activity of Gal-3C in the *in vivo* model may be mediated by interactions involving the tumor environment rather than by direct cytotoxicity to the MM cells is supported by our finding that the modest sensitivity of the MM cells to Gal-3C did not appear to depend on their galectin-3 expression levels.

The localization and retention of MM cells in the bone marrow is a hallmark of MM although the small numbers of MM cells found in the circulation are thought to represent the tumor-spreading component. The number of circulating cells increases at the end stage of disease, when MM cells are thought to gain the ability to proliferate outside of the bone marrow microenvironment and grow at extramedullary sites [39]. Thus, the processes of chemotaxis and invasion play a role in the pathophysiology of MM.

As shown in Figure 2a, Gal-3C at 2.0 µg/ml inhibited more than 60% of the U266 cell chemotaxis stimulated by the chemokine, SDF-1α. The SDF-1α and its receptor, CXCR4, are regulators of the migration and homing of MM cells to the bone marrow, and possibly may also control egression of MM cells out of the bone marrow [40]. Previously, galectin-3 was shown to induce the migration of monocytes, macrophages, and dendritic cells [41]. Modified citrus pectin is thought to act by binding galectin-3 and was shown to inhibit VEGF-induced chemotaxis of MM-1S MM cells when at the 200–400 µg/ml concentration [42].

Gal-3C at 10 µg/ml and Bor at 5 nM inhibited more than 30% of U266 cell invasion of Transwell chamber inserts with 5 µm pores that were coated with Matrigel as shown in Figure 2b. Invasion in this assay also was induced by SDF-1α in the bottom chamber. When Gal-3C was combined with Bor, more than 60% of the U266 cell invasion was inhibited. To our knowledge this is the first report that inhibition of galectin-3 can reduce the invasiveness of MM cells.

Angiogenesis plays a key role in the interactions between MM cells and their microenvironment [43]–[45], and recent data suggest that VEGF is the main mediator of MM-induced angiogenesis [46]–[48]. Galectin-3 has been shown to facilitate, and Gal-3C to inhibit VEGF-mediated angiogenesis [49]. Importantly, increased angiogenesis has been found to be indicative of poor prognosis in MM patients [45], [50], [51]. Thus, we postulated that the effects of Gal-3C *in vivo* could be at least partly due to inhibition of angiogenesis induced by the engrafted U266 cells, and tested this postulate using *in vitro* HUVEC migration and angiogenesis assays. Our results show that the media derived from U266 cells treated with Gal-3C in combination with Bor induced significantly less HUVEC migration and angiogenesis as revealed by tubule formation *in vitro* compared to media derived from untreated U266 cells (Figure 3). The single treatments significantly inhibited angiogenesis (tubule formation) but not HUVEC migration. We eliminated the possibility that the inhibition observed was due to reduced HUVEC viability, since Gal-3C did not display significant effects on HUVEC viability at the concentration used in the chemotaxis and angiogenesis assays, and Bor had an effect only at a concentration 5-fold higher (Figure 4). Furthermore, at the concentration used in HUVEC assays, Bor did not display any effect on HUVEC viability when combined with different concentrations of Gal-3C (Figure 4).

Because ανβ3 integrin engagement has been shown to be required for angiogenesis [52], we tested the ability of c.m. derived from differently treated U266 cells to induce ανβ3 integrin activation in HUVEC cells. Figure 5 shows that the combined, as well as the single treatments, effectively blocked ανβ3 integrin activation, as evidenced as protein clusters [49]. This result is in accordance with the observation that single and combined Gal-3C and Bor treatments were able to hamper tubule formation induced by U266 c.m. (Figure 3c).

ELISA assays showed that Gal-3C and Bor alone or combined significantly reduced U266 ability to secrete VEGF, but a significant decrease in bFGF levels was observed only with Bor, alone or combined with Gal-3C (Figure 6). These observations suggest that the ability of Gal-3C and Bor to hamper HUVEC migration and *in vitro* angiogenesis is at least in part due to down-regulation of VEGF and bFGF.

Gal-3C had more efficacy than Bor as a single agent when delivered intravenously using an osmotic mini-pump in NOD/SCID mice based on differences in tumor volume at the end of the study (d 35, *P*<0.001). Importantly for potential clinical applications, the combination of Gal-3C with Bor significantly reduced tumor growth (*P*<0.001) compared to treatment with either agent alone beginning from d 28.

In our previous study in a CD-1 nude mouse model of metastatic human breast cancer [17], Gal-3C (125 µg/dose) was injected intramuscularly twice daily whereas in the current study, approximately 30 µg/d was administered daily using an osmotic pump for continuous delivery. Our data suggest that sustained delivery may be preferable for maximal response to treatment, which is supported by our previous finding that the half-life of Gal-3C in mice was only approximately 3 h [17].

In addition to monitoring disease progression by measuring the tumors with calipers, the levels of human IgE and Igλ in the sera of tumor-bearing mice were analyzed by ELISA and found to be higher in comparison to control mice, consistent with the *in vivo* expansion of the U266 cells that secrete IgE [53]. Contemporaneously, AKAP-4, a member of scaffolding protein family, was used as a tumor marker. Heavy and light chain IgE and AKAP-4 levels were significantly lower in treated groups compared to the PBS group on d 21, 28, and 35. The levels of human IgE and Igλ in both the serum and tumor mass were lower in the Gal-3C plus Bor group compared with all other groups. These results support the differences observed between the groups from the tumor volume measurements. These results were also confirmed by cytofluorimetric analysis of IgE and Igλ on cells from tumor masses (Figure 10), and are in accordance with the increased levels of IgE and Igλ in the sera of MM patients that are used in diagnosis and prognosis [54]-[56].

Our prior study investigating AKAP-4 expression in MM and in normal tissues demonstrated that it is a novel MM tumor-associated antigen [57]. Both AKAP-4 and GFP were detected in all tumor masses. The expression of AKAP-4 and GFP was the lowest in animals treated with Gal-3C plus Bor, mirroring the results obtained with IgE and Igλ and caliper measurements. Moreover, the results obtained with the two different markers GFP and AKAP-4 were concordant, further confirming that AKAP-4 is a novel, reliable biomarker for MM.

These data demonstrate that serum levels of the human monoclonal Igs and AKAP-4 can be used to monitor MM progression *in vivo* in murine models and aid in the pre-clinical evaluation of new anti-myeloma therapies. An advantage of AKAP-4 for clinical use would be that the serum levels of the protein are normally very low in humans, unlike human Ig.

There is evidence that members of the galectin family other than galectin-3 [49], such as galectin-1 [58] and galectin-8 [59] induce angiogenesis and are tumorigenic. Galectin-1 is involved in cell cycle regulation, apoptosis, cell migration, and tumor evasion of immune responses [58], [60]. Galectin-8 promotes cell adhesion by binding cell surface integrins [61], and its levels have been correlated with cancer progression [61].

Tumor growth and metastasis were suppressed in mice lacking Mgat5, an enzyme forming antenna-like oligosaccharides containing lactosamines [62]. Mgat5-extended poly-*N*-acetyllactosamine (poly-LacNAc) binding sites typically have two or more LacNAcs [63]. As a dominant negative inhibitor, Gal-3C would block carbohydrate binding sites of some other members of the galectin family. For example, comparing dissociation constants determined by frontal affinity chromatography, Gal-3C has been more affinity for LacNAc2, (Kd = 4.8 µM), LacNAc3, (Kd = 0.87 µM) and LacNAc5, (Kd = 0.43 µM) than galectin-1 (Kd = 50, 45, and 39 µM, respectively) and similar or more affinity than galectin-8 (Kd = 3.5, 9.6 and 1.6 µM, respectively) [63].

The translational potential of our results is evident when considering that neovascularization of bone marrow is thought to support the progression of MM and that inhibition of angiogenesis is one of the key mechanisms of action of several current frontline drugs for MM therapy, such as thalidomide [64], [65], lenalidomide [66] and Bor itself [65], [67], indicating that inhibition of blood vessel formation might account for the synergistic effect of Gal-3C and Bor observed *in vivo*. Notably, it has been previously shown that galectin-3-mediated µ ανβ3-integrin clustering and activation in HUVEC cells are required for cell migration and angiogenesis [49], but this is the first time that galectin-3 has been reported to control the angiogenic potential of MM cells by modulating the expression of VEGF. The mechanism of the Gal-3C-mediated down-regulation of VEGF will be subject of future investigations.

The mechanism of action of Bor has been found to be more complex than originally thought. In MM cells specifically, Bor has been shown to induce the canonical NF-κB pathway by triggering IKKβ-mediated phosphorylation of IKBα [68]. This effect potentially reduces the therapeutic effect of Bor, because NF-κB activation in plasma cells is associated with anti-apoptotic pathways [68]. Accordingly, IKK inhibitors potentiate Bor cytotoxicity *in vitro* and the therapeutic combination of Bor with the IKK inhibitors PS-1145 and MLN120B has been proposed [68]. Previous reports show that galectin-3 can induce the NF-κB pathway and activation of downstream target genes [69], and that inhibition of galectin-3 with modified citrus pectin (GCS-100) [42] impairs TNF-α induced activation of NF-κB by hampering IKKα phosphorylation of MM cells [70]. In future studies we will examine the effect of Gal-3C on the Bor-mediated induction of the NF-κB pathway in MM cells.

An advantage of our approach is that Gal-3C appears to have a low toxicity profile [17] but is nonetheless capable of chemosensitizing MM cells. Our work indicates that Gal-3C can inhibit the proliferation, chemotaxis, and invasiveness of MM cells, and MM-cell induced angiogenesis, and provides the first evidence of the effectiveness of Gal-3C in combination therapy *in vivo* in an animal model of human MM where we also showed that Gal-3C was more effective than Bor as a monotherapy. Our data indicate that Gal-3C potentially may help to fulfill the need for new targeted therapies for the treatment of MM [71]. The significance of our approach is highlighted by recent findings that agents able to block the vicious cycle generated by the complex interactions between MM cells and their microenvironment may prove to be a key in future management of this still incurable disease [71]. Gal-3C has previously shown anticancer activity in a mouse model of metastatic breast cancer, and is thought to act through inhibition of functionality of galectin-3 which has been strongly implicated in the progression of cancer. Our results provide proof-of-principle for the activity of Gal-3C in MM that justifies continued investigation aimed at the initiation of clinical testing.

## Materials and Methods

### Animals

Six-week-old female NOD.CB17-Prkdcscid/J (NOD/SCID) mice were obtained from the Jackson Laboratory (Bar Harbor, ME, USA). All mice were maintained in filtered-air laminar-flow cabinets under specific pathogen-free conditions. Treatment and care were in accordance with the Institutional Guidelines of the Texas Tech University Laboratory Animal Resources Center (LARC) and the Animal Welfare Assurance Act. All mice were euthanized before they were 10 weeks of age.

### Cells and media

The human MM cell lines used in this study were RPMI-8226, U266 (American Type Culture Collection, Manassas, VA, USA), ARP-1, ARK-B, (gifts from J. Epstein, University of Arkansas for Medical Sciences, Little Rock, AR, USA). The MM cell lines MM.1S and MM.1RL were generously provided by Dr. Steven T. Rosen, of Northwestern University in Evanston, Illinois [72], and the NCI-H929 cell line was donated by Dr. Steven D. Rosen of the University of California, San Francisco. The 8226/Dox and 8226/LR-5 cell lines were kindly provided by Dr. William S. Dalton of the H. Lee Moffitt Cancer Center and Research Institute in Tampa, Florida [73]. All MM cells were cultured in RPMI-1640 medium supplemented with 10% fetal bovine serum (FBS) in 5% CO_2_ at 37°C. The 8226/Dox cells were cultured with 40 nM doxorubicin (Sigma-Aldrich, St. Louis, MO, USA). The 8226/LR-5 cells were cultured in media containing 5 µM mephalan (Sigma-Aldrich). 8226/Dox and 8226/LR-5 cells were maintained in drug-free medium for 1 week prior to drug sensitivity assays. HUVEC (American Type Culture Collection) were maintained in EGM-2 medium (Lonza, Houston, TX, USA) and were used within 10 passages.

### SDS-PAGE and immunoblot

MM cells were harvested and washed twice with PBS. Cell lysis buffer was prepared by mixing 2 ml of M-PER protein extraction buffer (Pierce/Thermo, Rockford, IL, USA) with 40 µl of Protease Inhibitor Cocktail (Sigma-Aldrich). The cell suspensions were transferred to a new tube, homogenized using a handheld mortar and pestle, and then rocked at room temperature (RT) for 10 min. Extracts were clarified by centrifuging at 12,000×RPM for 15 min, diluted to a final concentration of 2.5 mg/ml protein using M-PER, LDS buffer (Invitrogen, Carlsbad, CA, USA), and the reducing agent, 2-mercaptoethanol, and then heated at 70°C for 10 min. Proteins were resolved on 4–12% Bis-Tris (Bis(2-hydroxyethyl)-amino-tris(hydroxymethyl)-methane) polyacrylamide gel (Invitrogen) and then electrotransferred onto 0.2 um nitrocellulose membrane. After rinsing in PBS-Tween, the blot was stored in protein-free blocking buffer (Pierce/Thermo) at 4°C overnight. Then, the blot was incubated with anti-galectin-3 antibody [74] (M3/38, Santa Cruz Biotechnology, Santa Cruz, CA, USA) diluted 1∶200 in blocking buffer at RT for 30 min. After washing 5 times with PBS-Tween, the blot was incubated with biotin-conjugated anti-rat IgG diluted 1∶2000 in blocking buffer at RT for 1 h. After 5 washings, the blot was incubated with horseradish peroxidase-conjugated streptavidin diluted 1∶1000 in blocking buffer at RT for 20 min, and then developed with 3,3′,5,5′-tetramethylbenzidine (TMB) with membrane enhancer (KPL; Mandel Scientific, Guelph, Canada). Images were captured with a digital camera and digitally enhanced (Adobe PhotoShop or ImageJ).

### Drugs and mini-pumps

Gal-3C was prepared as previously described [17]. Bor was purchased from Millennium Pharmaceuticals (Cambridge, MA, USA). Doxorubicin and mephalan were obtained from Sigma-Aldrich. The 200-µL 2002 mini-osmotic pumps designed to deliver 0.5 µL/h were purchased from DURECT Corporation (Cupertino, CA, USA).

### Cell proliferation and viability

Cell proliferation was assessed with a ViaLight Plus Cell Proliferation and Cytotoxicity BioAssay Kit (Lonza, Walkersville, MD, USA) according to the directions of the manufacturer. In brief, myeloma cells were seeded (8,000 per well) in 50 µl of RPMI-1640 with 10% heat-inactivated FBS (growth medium) in 96-well plates. HUVEC cells were seeded (10,000/well) in 50 µl EGM-2 medium in 96-well plates. Gal-3C (2X concentration) in 50 µL of growth medium was added to triplicate wells and the plate kept at the CO_2_ incubator at 37°C for 24 or 48 h. For the ViaLight assay, 50 µL of lysis buffer was added to each well, the plate was incubated for 10 min, after which 50 µL of cell lysate from each well was transferred to a solid white Lumitrac-200 plate and 50 µL of reconstituted AMR Plus was added. Luminescence was measured with a 1-s integrated setting in a Berthold luminometer after 2 min. With Alamar Blue, 10 µL of reagent was added per well and the plate kept in a CO_2_ incubator for 4 h. Fluorescence intensity was measured at 590-nm on a Polarstar Galaxy (BMG Labtech, Cary, NC, USA) microplate reader. The blank (growth media with 10 µl Alamar Blue) was subtracted from the raw fluorescence to obtain adjusted fluorescence readings.

### Chemotaxis assays

U266 cells (400×10^3^/well) were plated in 100-µl cell culture media with and without Gal-3C (0, 0.4, 2.0, 10, and 20 µg/ml) in the top chambers of 24-well transwell inserts with 8-µm pores. Cell culture medium (600 µl) containing the B-cell chemoattractant SDF-1α (100 ng/ml; R&D Systems, Minneapolis, MN, USA) was added to the bottom chamber. After 4 h, the U266 cells in the bottom chamber were counted with a FACScan flow cytometer (flux rate on high, a 2 min acquisition gate, and 200 µs resolution).

For HUVEC cells, chemotaxis was analyzed as reported [49], using 50×10^3^ cells/well and 24-well transwell inserts with 8-µm pores. The lower chamber was filled with 600-µl EGM-2 medium supplemented with 20% V/V c.m., obtained after treatment of U266 cells with 10 µg/mL Gal-3C, 5 nM Bor, or 10 µg/mL Gal-3C with 5 nM Bor for 48 h, or with 0.6% V/V PBS (vehicle) as a control. As a negative control, EGM-2 medium alone was used. After incubation for 16 h, cells were removed from the upper chamber, and the migrated cells were visualized on the lower side of the filter after fixing and staining with 30% methanol, 10% acetic and 0.1% Coomassie Brilliant Blue. Next, the filters were extensively washed with water, and then air-dried and photographed using an inverted X71 microscope (Olympus, Center Valley, PA, USA). The average number of cells in 4 random fields per filter was calculated. The experiments were run in triplicate and the results are expressed as a migration index (MI), as follows: MI = number of cells migrated in the presence of c.m./number of cells migrated in the presence of EGM-2 alone.

### Invasion assay

An invasion assay was performed with U266 cells in 24-well, 6.5-mm internal diameter Transwell® chamber plates with polycarbonate membranes (5-µm pore size, Corning Costar, NY) as described previously [28]. Briefly the upper chambers were coated with 50-µl Matrigel (Becton Dickinson, Bedford, MA, USA) in advance according to the directions of the manufacturer. U266 cells (5×10^4^) in suspension were starved in serum-free RPMI-1640 for 3 h, and then loaded onto the Matrigel-coated inserts in the upper chambers. The lower chamber of the transwell was filled with 600-µl of 10% FBS-containing cell culture media with 100 ng/ml SDF-1α. Gal-3C (10 µg/ml), Bor (5 nM), Gal-3C with Bor, or PBS vehicle-only were added with the cells to the upper chamber. At the 5 nM concentration used, the cytotoxicity of Bor for the U266 cells was expected to be minimal [75]. Plates were then incubated at 37°C for 24 h. At the end of the incubation period, non-invasive cells on the top of the transwell were scraped off with a cotton swab. Cells that had invaded the bottom chamber were fixed in methanol, stained with Coomassie, and then counted using a light microscope as described above for the chemotaxis assay.

### Tubule formation assay

A tubule formation assay was performed *in vitro* as described [49] to measure angiogenesis. Briefly, growth factor–reduced Matrigel (50 µL/well; Becton Dickinson) was allowed to polymerize in the presence of 30 ng/mL recombinant basic fibroblastic growth factor (bFGF; R&D Systems) for 1 h at 37°C. HUVECs (5,000/well), serum-starved for 2 h prior to trypsinization, were resuspended in 200-µL serum-free EGM-2 supplemented with 20% V/V c.m. obtained after treatment of U266 cells with 10 µg/mL Gal-3C, 5 nM Bor, the combination treatment, or 0.6% V/V PBS (vehicle) as a control for 48 h. The positive control was EGM-2 with 20 ng/mL VEGF (R&D Systems) [76], while the negative control was EGM-2 alone. Cells were seeded onto the polymerized Matrigel and incubated at 37°C with 5% CO_2_ for 16 h to allow tubule formation. The assays were run in triplicate, and microphotographs were taken with an inverted X71 microscope (Olympus).

### αµβ3 Integrin cluster formation assay

HUVECs were cultured in 8-chamber slides coated with 10 µg/ml fibronectin (10^4^ cells/chamber). Cells were incubated with c.m. for 20 min, and fixed with 4% paraformaldehyde in PBS (10 min at 37°C). Then, cells were incubated with 10 µg/ml anti-integrin αvβ3 antibody (R&D Systems, 1∶200 dilution in PBS) for 1 h at RT, followed by the addition of FITC-anti–mouse IgG (1∶1000 dilution in PBS) for 1 h at RT. Microphotographs of randomly selected fields were acquired at 60X magnification by confocal microscopy.

### ELISA for VEGF and bFGF

The levels of VEGF and bFGF were measured by direct ELISA as follows. Flat-bottom 96-well polycarbonate plates were coated at 4°C with 50 µL/well cell culture supernatants diluted 1∶25 in carbonate coating buffer (0.1 M Na_2_CO_3_, 0.1 M NaHCO_3_, pH 9.5) at 4°C overnight. Standard curves were obtained with purified recombinant human VEGF and bFGF (both from R&D Systems) serially diluted in coating buffer (10-fold serial dilutions from 1,000 to 0 ng/mL). After removing diluted supernatants or standards, and blocking with PBS supplemented with 1% W/V BSA for 1 h at RT, plates were incubated with mouse anti-human VEGF or rabbit anti-human bFGF primary antibodies (2 µg/mL in PBS, 50 µL/well Abcam, MA, USA) for 1 h at RT. Then, plates were washed twice with PBS with 0.025% V/V Tween-20 (300 µL/well) and incubated at RT with anti-mouse or anti-rabbit HRP-labeled secondary antibodies (0.2 µg/mL in PBS, 50 µl/well; Abcam, Cambridge, MA, USA) for 1 h. The plates were washed thrice with PBS containing 0.025% V/V Tween-20 (300 µL/well), the TMB colorimetric substrate (Thermo Fisher Scientific, Waltham, MA , USA) was added, and after 5 min the absorbance was measured (0.1 s) at 450 nm on a Victor 2 multimodal microplate reader (PerkinElmer, Billerica, MA, USA). All samples were run in triplicates.

### Generating AAV/GFP genome and virus stocks

The adeno-associated virus/green fluorescent protein (AAV/GFP) genome was constructed as follows. Briefly, AAV/GFP virus stocks were generated using complementary plasmids in s96-0.8 or pSH3 in HEK293 cells. Lysates of HEK293 cells were used as virus-negative control for mock infections. The DNA was extracted from AAV/GFP virus crude lysates as the sample for titer testing. The titers of virus stocks were determined by real-time PCR. Briefly, we used the plasmid AAV/GFP for the real-time PCR standards. Concentration was measured by absorbance at 260 nm. The real-time PCR was performed on an ABI Prism 7000 instrument (Applied Biosystems, Darmstadt, Germany) in a 50-µl reaction volume. Twenty µl of master PCR mix was combined with 10-µl primers. Thermal cycling conditions were as follows: 95°C for 10 min, 45 cycles of 95°C for 15 seconds, and 55°C for 1 min.

### Testing Gal-3C and Bor in NOD/SCID subcutaneous mouse model of MM

Prior to injection, U266 cells were washed once in Dulbecco's PBS (Sigma-Aldrich), counted, and adjusted to the appropriate density with additional PBS. The suspension containing 1×10^7^ U266 cells was injected subcutaneously into the abdomen of naive NOD/SCID mice. Primary tumors were measured with a pair of calipers once a week. Tumor volume was calculated by the formula width^2^×length×0.5, where width was the smallest dimension.

The mice were randomly divided into four groups with five mice in each group after 14 d. Control group #1 was subcutaneously implanted in the abdomen with a PBS-only containing 200-µl mini-osmotic pump. Group #2 received a dose of Gal-3C (30 µg/d/mouse) subcutaneously over a 16-d period for a total of 500 µg via a 200-µl mini-osmotic pump implanted abdominally. Group #3 was treated with six doses of 15 µg Bor in 50-µl injections via the tail vein on d 1–2, 8–9, and 15–16 for a total of 90 µg. Group #4 received Gal-3C via mini-pump for a total dose of 500 µg Gal-3C (30 µg/d/mouse) plus a total 90 µg Bor in 21 d. Anesthetized animals were euthanized and a post-mortem examination was conducted on the whole animals and dissected organs after 35 d.

### Real-time PCR

Total RNA was extracted from the U266 cells, and cells from tissue of the tumors, spleen, kidney, stomach, heart, lung and liver by means of a Trizol-reagent (Sigma-Aldrich) and isolated with the Oligotex mRNA Mini Kit (QIAGEN, Valencia, CA, USA), after DNase I digestion. First-strand cDNA synthesis was performed using oligo (dT) 15 primers. PCR primers were as follows: 5′-GCGTACTCTGATACTACAATGATG-3′ and 5′-GGG GTTTTGGGTAAAGTCA-3′ for AKAP4; and 5- CGGTCGCCACCATGGTGAGC-3′ and 5′-GAGCCGTACCTGCTCGACATG-3′ for GFP. The sizes of the primers were amplified for AKAP4 to 1100 base pairs (bp) and for GFP to 730 bp, respectively. Positive control was the cDNA of the U266 cells, and negative control was the PCR reaction mixture with water in place of cDNA. RNA integrity in each sample was checked by amplification of a β-actin gene segment.

### ELISA for human AKAP-4, IgG, E, k, and λ light chain

Blood was collected from each mouse once a week, and an ELISA was performed on the sera to quantify human AKAP-4, IgG, IgE, κ, and λ light chain levels as reported [77]. Antibodies for Igs were purchased from BD Biosciences. Anti AKAP-4 antibody was from Santa Cruz Biotechnology. Signal intensity was measured on a Victor 2 multimodal microplate reader (PerkinElmer, MA, USA) at 450 nm. All samples were run in triplicates.

### Flow cytometric analysis

The expression of AKAP-4, human IgE, IgG, (heavy chain) κ, and λ light chains was analyzed by cytofluorimetric techniques. Briefly, cells from liver, blood, and tumor masses of animals from each group were minced under sterile conditions at RT to obtain single-cell suspensions. Minced tumor tissues were placed into 250-ml flasks containing 3-ml of an enzyme solution that consisted of 0.14% collagenase type I (Sigma-Aldrich) and 0.01% DNase (2000 kU/mg of tissue; Sigma-Aldrich) in RPMI 1640, and then incubated on a magnetic-stirring apparatus at 37°C for 30 min. Dissociated tissues were filtered through a 150-µm pore-size nylon mesh to generate a single-cell suspension which was washed twice in RPMI 1640 supplemented with 10% FBS and penicillin/streptomycin.

Cell suspensions were distributed into 12×75 mm tubes (5×10^5^ cells/tube). Cells were incubated with monoclonal antibodies raised against human Ig and AKAP-4 or isotype matching antibodies (BD Biosciences, CA, USA) as negative controls. Analysis was performed using a fluorescence-activated cell scanner (BD Biosciences).

### Statistical analyses

All of the data are expressed as mean values, and as indicated either±the standard error of the mean (SEM) or standard deviation (S.D.). Results were analyzed using GraphPad Prism version 4.00 for Windows (GraphPad Software, San Diego, CA, USA). Univariate analyses were performed using a Student's t test as required for parametric variables with *P*<0.05 as the significance limit.

## Supporting Information

Figure S1 **Viral vector used in the study and analysis of infection.** (A) A map of the AAV/GFP expression vector is presented. The restriction sites Xba I and Not I were used to insert the GFP coding sequence. (B) The ligation of AAV genome and GFP is shown by restriction digestion analysis. The GFP insert was cut out from AAV/GFP by Xba I and Not I (lane 2). (C) Quantification of virus titers by real-time PCR. DNA was prepared from 1000-µl, 500-µl and 250-µl volumes of the cell crude lysates of AAV/GFP-infected HEK293 cells and used as template for real-time PCR. The titer was 10^8^ encapsulated genomes/ml. (D) Identification of U266 cells transduced with AAV/GFP by flow cytometry. Grey and black histograms indicate the fluorescence intensity distribution of AAV/GFP- and AAV-transduced U266 cells, respectively. (E) RT-PCR for GFP expression in transduced U266 cells. M: Marker, 1: AAV/GFP plasmid, 2: No RT, 3: No template, 4: U266 cells transfected with AAV/GFP. The size of the band from lane 4 was the same as the lane 1. (F) Photomicrograph of U266 cells showing fluorescence of AAV/GFP-transduced U266 cells.
(TIF)
