# Supplementary figures and images for: Galectin-3C Inhibits Tumor Growth and Increases the Anticancer Activity of Bortezomib in a Murine Model of Human Multiple Myeloma
Source: PLoS One. 2011 Jul 13;6(7):e21811. doi: 10.1371/journal.pone.0021811 (PMC3135605; doi:10.1371/journal.pone.0021811)

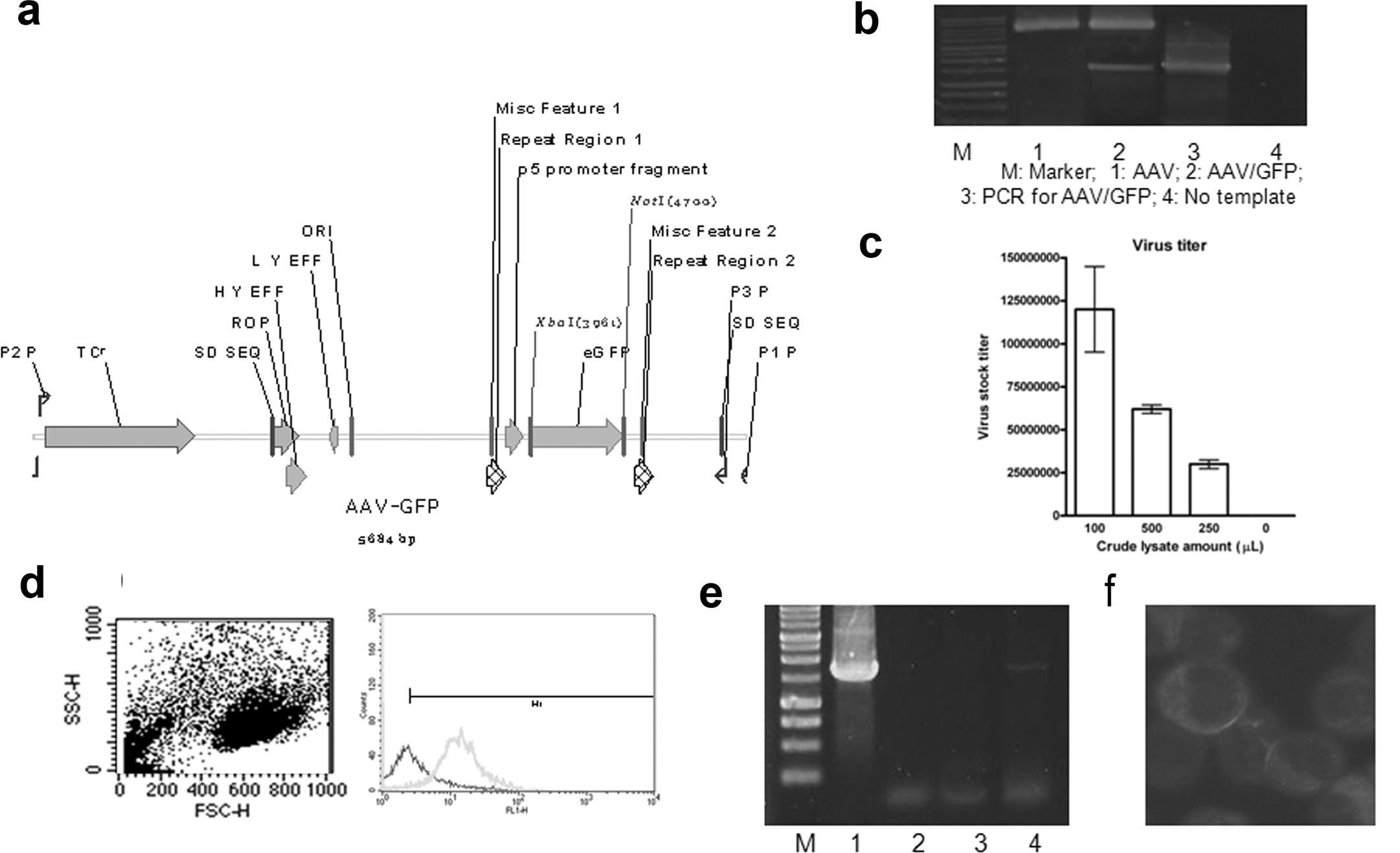

Supplement: Figure S1 — Viral vector used in the study and analysis of infection. (A) A map of the AAV/GFP expression vector is presented. The restriction sites Xba I and Not I were used to insert the GFP coding sequence. (B) The ligation of AAV genome and GFP is shown by restriction digestion analysis. The GFP insert was cut out from AAV/GFP by Xba I and Not I (lane 2). (C) Quantification of virus titers by real-time PCR. DNA was prepared from 1000-µl, 500-µl and 250-µl volumes of the cell crude lysates of AAV/GFP-infected HEK293 cells and used as template for real-time PCR. The titer was 108 encapsulated genomes/ml. (D) Identification of U266 cells transduced with AAV/GFP by flow cytometry. Grey and black histograms indicate the fluorescence intensity distribution of AAV/GFP- and AAV-transduced U266 cells, respectively. (E) RT-PCR for GFP expression in transduced U266 cells. M: Marker, 1: AAV/GFP plasmid, 2: No RT, 3: No template, 4: U266 cells transfected with AAV/GFP. The size of the band from lane 4 was the same as the lane 1. (F) Photomicrograph of U266 cells showing fluorescence of AAV/GFP-transduced U266 cells. (TIF) [file pone.0021811.s001.tif]
